# Association of Gender With Outcomes in Hospitalized Patients With 2019-nCoV Infection in Wuhan

**DOI:** 10.3389/fpubh.2021.619482

**Published:** 2021-06-15

**Authors:** Huiwu Han, Xiaobei Peng, Fan Zheng, Guiyuan Deng, Xiaocui Cheng, Liming Peng

**Affiliations:** ^1^Teaching and Research Section of Clinical Nursing, Xiangya Hospital of Central South University, Changsha, China; ^2^Xiangya School of Nursing, Central South University, Changsha, China; ^3^Critical Care Medicine Department, Xiangya Hospital at Central South University, Changsha, China; ^4^Health Management Department, Xiangya Hospital at Central South University, Changsha, China; ^5^Cardiovascular Medicine Department, Xiangya Hospital at Central South University, Changsha, China; ^6^Ophthalmology Department, Union Hospital, Huazhong University of Science and Technology, Wuhan, China

**Keywords:** 2019-nCoV, gender, disease outcome, psychological situation, smoking

## Abstract

**Aim:** The aim of this study was to analyze the association of gender with psychological status and clinical outcomes among patients with 2019-nCoV infection to provide new directions for the prevention and control of the pandemic.

**Methods:** One hundred and thirty-eight patients with confirmed 2019-nCoV infection at Wuhan Union Hospital, between February 8 and March 31, 2020, were included in the study analysis. General information and data on clinical characteristics were collected from patients' medical records. Participants' responses to self-report measures of psychological status were also collected.

**Results:** Anxiety levels, depression levels, and recovery rates were significantly higher among women compared to men. Conversely, chronic disease history and smoking rates, dry cough incidence, C-reactive protein levels, and disease severity were significantly higher among men than women (*p* < 0.05).

**Conclusion:** Female patients experienced more severe psychological issues, due to higher levels of anxiety and stress, than male patients; indicating that more attention should be paid to the psychological care of female patients. In contrast, the general condition of male patients was more severe, particularly among elderly male patients with a history of chronic disease and smoking, suggesting that, to prevent and control 2019-nCoV infection, male patients should be encouraged to quit smoking as soon as possible to reduce the risk of severe pneumonia.

## Introduction

On December 8, 2019, several cases of unexplained pneumonia were reported among patients with a history of contact with the Huanan seafood market, in the city of Wuhan, China. These patients further presented symptoms of severe acute respiratory infection, quickly developing into acute respiratory distress syndrome (ARDS) and acute respiratory failure, which were later confirmed to be caused by a novel corona-virus (1). On January 7, 2020, this novel corona-virus was isolated and identified in a sample from a patient's throat swab by the Chinese Center for Disease Control and Prevention (China CDC). The World Health Organization subsequently named it as corona-virus disease 2019 (2019-nCoV).

2019-nCoV was first identified in China; however, countries in North and South America and Europe have been most affected by the virus. The virus spread quickly and became a global public health emergency. At present, there are more than 153 million confirmed cases of 2019-nCoV around the world, and more than 3.2 million related deaths have been recorded. In the current public health emergency, it is imperative to understand the epidemiological and clinical features of the 2019-nCoV infection. Catastrophic events and their devastating consequences are unforeseeable and unavoidable. The psychological impact of such events on the population includes fear, anxiety, depression, stress, and sleep problems, among other issues (2). High levels of fear and anxiety have significant impact on patients diagnosed with 2019-nCoV, leading to psychological complications and influencing the effectiveness of treatment. Therefore, attention to patients' psychological status is an important part of effective treatment.

Gender differences in the severity and psychological impact of the 2019-nCoV infection have not been well-researched thus far. Understanding the gender differences associated with susceptibility and vulnerability toward 2019-nCoV infection is important to respond effectively to the public health emergency and minimize the health, economic, and social effects of the pandemic. This study explored the epidemiological and clinical features of 138 hospitalized patients with confirmed 2019-nCoV infection. Patient health questionnaire-9 (PHQ-9) and generalized anxiety disorder scale-7 (GAD-7) were used to the assess the patients' depression level and anxiety level, respectively. The clinical classification of 2019-nCoV infection and MulBSTA score were used to identify the severity of 2019-nCoV infection. The associations among psychological status, clinical outcomes, and patient gender were analyzed to inform psychological and therapeutic intervention for the prevention and control of the 2019-nCoV pandemic.

## Materials and Methods

### Study Design and Subjects

A retrospective, single-center study was conducted. One hundred sixty patients with confirmed 2019-nCoV infection were recruited from the three specialist wards of “2019-nCoV” in the west campus of Wuhan Union Hospital, a local hospital in Wuhan, between February 8, 2020 and March 31, 2020. Inclusion criteria was: (1) age ≥18 years; (2) with a positive 2019-nCoV nucleic acid test result by the reverse transcription polymerase chain reaction (RT-PCR) method; (3) met the diagnostic criteria of the Guidelines for the Diagnosis and Treatment of Novel Corona-virus (2019-nCoV) Infection (Trial Version 5) released by the Chinese National Health Commission (3); (4) hospital stay duration ≥ 2 weeks. Exclusion criteria was: communication barriers or the consciousness disorder due to disease severity. The study was conducted in accordance with the Declaration of Helsinki and approved by the Ethics Committee of Xiangya Hospital of Central South University (Approval No. 202003049; Date:04/22/2020). All the participants in the study were informed and signed the relevant consent forms.

### Data Collection

General information and data on the clinical characteristics of the participating 2019-nCoV infection patients were collected from the patients' medical record. General information included gender, age, education level, smoking history, chronic disease history and infectious disease history were collected after admitted to the hospital. Data on the clinical characteristics of the 2019-nCoV infection included incubation period, pulse oxygen saturation (SPO2), clinical symptoms, related complications, laboratory reports, radiologic features, clinical classification of 2019-nCoV infection and MulBSTA score were also collected after admitted to the hospital, and used the first data after hospitalization. Patients' depression levels were assessed using PHQ-9, and their anxiety levels were assessed using GAD-7 within 3 days after hospitalization, used the first data after hospitalization. The treatment effect (discharge, death, and continued hospitalization) and hospital stay were collected at discharge.

The PHQ-9 is a self-report scale of depression consisting of nine items (4). Subjects were asked to rate each item on a scale of 0–3 on the basis of how much a symptom has bothered them during the last 2 weeks (0 = not at all, 1 = several days, 2 = more than half the days, 3 = nearly every day). A total score ranging from 0 to 27 by summing up the scores from each item. The higher the score, the more severe the depression level. A cut-off score of 5 or above on the summed-item score was recommended as depressive disorder. The total score ≥ 10 was believed as moderate depression, and ≥ 15 was considered as severe depression. The Cornbach' α coefficient of PHQ-9 scale is 0.832, the test-retest reliability is 0.934, the sensitivity is 88%, and the specificity is 99% (5).

The GAD-7, consisting of 7 items, is a self-rated scale used for screening anxiety disorder. Items are rated on a 4-point scale (0 = not at all, 1 = several days, 2 = more than half the days, 3 = nearly every day). The total score ranges from 0–21 with higher scores presenting more severe anxiety disorder. A cut-off score of 5 or above on the summed-item score was recommended as anxiety disorder. The total score ≥ 10 was believed as moderate anxiety, and ≥ 15 was considered as severe anxiety. The Cornbach' α coefficient of GAD-7 scale is 0.898, the test-retest reliability is 0.856, the sensitivity is 86%, the specificity is 96%, and Kappa value is 0.825 (6).

The clinical classification of 2019-nCoV infection was divided to three types: mild, severe and critical cases, in accordance with “Guidelines for the Diagnosis and Treatment of Novel Corona-virus (2019-nCoV) Infection (Trial Version 5).” MulBSTA scale (7) was used in the study to assess the illness severity of 2019-nCoV pneumonia: imaging multilobe infiltration (5 scores); lymphocyte ≤ 0.8^*^10^9^/L (4 scores); bacterial infection (4 scores); acute-smoker (3 scores); former-smoker (2 scores); hypertension (2 scores); age ≥ 60 years (2 scores). The higher score, the more severe the pneumonia.

### Statistical Analysis

SPSS25.0 statistical software was applied for data analyzing. Measurement data conforming to a normal distribution was expressed as mean ± standard deviation ($\bar{x}$±*S*D), while *t*-test and Mann-Whitney U-test were used to compare the difference between the groups. Enumeration data was described as case numbers and percentages, and χ^2^ test was employed to compare the differences between gender groups. A two-sided *p* < 0.05 was considered statistically significant.

## Results

### Subjects Characteristics

In the study, a total of 160 patients with confirmed 2019-nCoV infection were recruited, and 22 cases were excluded from the analysis due to missing data. Among the 138 valid subjects, 59 were female (42.8%). The mean age of the subjects was 62.32 ± 11.19 years old, and 93 cases (67.4%) were ≥ 60 years old. Overall, 27 cases (19.6%) had a history of smoking, 46 cases (33.3%) had hypertension, 28 cases (20.3%) had coronary artery disease (CAD), and 26 cases (18.8%) had diabetes. Regarding the transmission route, a large cohort (61.6%) were unclear of the source of transmission of their infection. The most common clinical symptoms among patients were fever (64.5%), dry cough (58.7%), and dyspnea (26.8%). Complications, such as respiratory distress, bacterial infection, and respiratory failure, occurred in 50.7, 10.9, and 2.2% of the subjects, respectively. Most subjects (91.3%) were classified as severe and critical cases. By the end of this study, 110 subjects (79.7%) had recovered and were discharged from the hospital. The average duration of hospital stay was 28.08 ± 13.03 days (see Tables 1–**3**).

**Table 1:** Gender differences in general characteristics of 2019-nCoV infection.

**Variables**	**Total (*n* = 138)**	**Male (*n* = 79)**	**Female (*n* = 59)**	**t/X^**2**^ value**	***p*-value**
Age (years)	62.32 ± 11.19	62.26 ± 9.72	62.14 ± 12.98	0.166	0.869
Academic degree *n* (%)				0.599	0.439
≤ Junior school	79 (57.2)	43 (54.4)	36 (45.6)		
≥High school	59 (42.8)	23 (39.0)	36 (61.0)		
Smoking *n* (%)				20.914	0.000
Yes	27 (19.6)	26 (32.9)	1 (1.7)		
No	111 (80.4)	53 (67.1)	58 (98.3)		
**Chronic disease history** ***n*** **(%)**					
HBP	46 (33.3)	30 (38.0)	16 (27.1)	1.791	0.181
CAD	28 (20.3)	14 (17.7)	14 (23.7)	0.754	0.385
DM	26 (18.8)	15 (19.0)	11 (18.6)	0.003	0.959
Transmission route *n* (%)				0.225	0.635
Exposure history	53 (38.4)	29 (36.7)	24 (40.7)		
Unknown cause	85 (61.6)	50 (63.3)	35 (59.3)		

*HBP, hypertension; CAD, coronary artery disease; DM, diabetic mellitus*.

### Gender Differences in General Characteristics

Results indicate that smoking rates were significantly higher among men (32.9%) than women (1.7%; *p* < 0.05). No significant differences were found between male and female participants in their age, education level, chronic disease history (i.e., hypertension, CAD, and diabetes, among others), and source of transmission (see Table 1).

### Gender Differences in Clinical Characteristics

Results show that dry cough incidence was significantly higher in male patients (67.1%) than in their female counterparts (47.5%). The rate of increased C-reactive protein levels was also significantly higher in male patients (50.6%) compared to female patients (28.8%; *p* < 0.05) (see Table 2).

**Table 2:** Gender differences in clinical characteristics of 2019-nCoV infection.

**Variables**	**Total (*n* = 138)**	**Male (*n* = 79)**	**Female (*n* = 59)**	**Z/X^**2**^ value**	***p-*value**
Incubation period (days)	9.19 ± 5.18	9.73 ± 5.32	8.44 ± 4.94	−1.380	0.168
**Symptom** ***n*** **(%)**					
Fever	89 (64.5)	52 (65.8)	37 (62.7)	0.143	0.706
Dry cough	81 (58.7)	53 (67.1)	28 (47.5)	5.369	0.020
Dyspnea	37 (26.8)	21 (26.6)	16 (27.1)	0.005	0.944
Asthenia	36 (26.1)	22 (27.8)	14 (23.7)	0.297	0.586
SPO_2_(%)	95.06 ± 4.59	94.85 ± 3.97	95.34 ± 5.34	−0.870	0.384
**Complication** ***n*** **(%)**					
Respiratory distress	70 (50.7)	45 (57.0)	25 (42.5)	2.876	0.090
Bacterial infection	15 (10.9)	9 (11.4)	6 (10.2)	0.052	0.819
Respiratory failure	3 (2.2)	2 (2.5)	1 (1.7)	0.111	0.739
**Laboratory result** ***n*** **(%)**					
Normal or decreased WBC count	134 (97.1)	77 (97.5)	57 (96.6)	0.088	0.766
Decreased lymphocyte count	55 (39.9)	32 (40.5)	23 (39.0)	0.033	0.857
Increased C-reactive protein level	57 (41.3)	40 (50.6)	17 (28.8)	6.633	0.010
Increased procalcitonin	48 (34.8)	32 (40.5)	17 (28.8)	2.770	0.250
Imaging multilobe infiltration *n* (%)	135 (97.8)	79 (100)	56 (94.9)	4.106	0.128

*SPO_2_, oxygen saturation; WBC, white blood cell*.

### Gender Differences in Psychological Status and Clinical Outcomes

Results of the analysis of gender differences in participants' psychological status (based on PHQ-9 and GAD-7 scores) and clinical outcomes (based on 2019-nCoV infection classification, MulBSTA score, treatment effect, and hospital stay duration) show that the anxiety levels, depression levels, and recovery rates of female patients were significantly higher than male patients. Conversely, disease severity scores (based clinical classification and MulBSTA score) was significantly higher in male patients than in the female patients (*p* < 0.05) (see Table 3). Results of multivariate logistic regression analysis on the clinical classification of 2019-nCoV further confirm that there was significant difference between genders in the participants with severe 2019-nCoV infection (see Table 4).

**Table 3:** Gender differences in psychological status and clinical outcomes of 2019-nCoV infection.

**Variables**	**Total (*n* = 138)**	**Male (*n* = 79)**	**Female (*n* = 59)**	**Z/X^**2**^ value**	***p*-value**
PHQ-9 score (±*S*D)	5.74 ± 4.95	4.52 ± 4.40	7.31 ± 5.20	−3.165	0.002
GAD-7 score (±*S*D)	4.23 ± 4.35	3.32 ± 3.93	5.40 ± 4.62	−2.899	0.004
Clinical classification *n* (%)				6.605	0.037
Mild	12 (8.7)	4 (5.0)	8 (13.6)		
Severe	110 (79.7)	62 (78.5)	48 (81.4)		
Critical	16 (11.6)	13 (16.5)	3 (5.0)		
MuLBSTA score (±*S*D)	8.04 ± 3.25	8.87 ± 3.48	6.91 ± 2.50	−3.360	0.001
Treatment effect *n* (%)				7.157	0.028
Discharge	110 (79.7)	57 (72.2)	53 (89.8)		
Death	3 (2.2)	3 (3.8)	0 (0.0)		
Continued hospitalization	25 (18.1)	19 (24.0)	6 (10.2)		
Hospital stay (days)	28.08 ± 13.03	28.78 ± 12.45	27.31 ± 13.72	0.661	0.508

**Table 4:** Multivariate logistic regression analysis on the clinical classification of 2019-nCoV.

**Variables**	**Severe**			**Critical**		
	**β**	**OR (95%CI)**	***p***	**β**	**OR (95%CI)**	***P***
Gender (0.female,1.male)	1.577	4.842 (1.066–22.006)	0.041	1.701	5.478 (0.536–56.027)	0.152
Smoking (0.no,1.yes)	−0.470	0.625 (0.095–4.135)	0.626	1.366	3.921 (0.386–39.844)	0.248
PHQ-9 score	0.096	1.100 (0.888–1.364)	0.382	0.131	1.140 (0.847–1.534)	0.387
GAD-7 score	0.120	1.128 (0.875–1.452)	0.352	0.004	1.004 (0.702–1.437)	0.981

*OR, odds ratio. The clinical classification of 2019-nCoV infection was divided to three types: mild, severe and critical cases, in accordance with “Guidelines for the Diagnosis and Treatment of Novel Corona-virus (2019-nCoV) Infection (Trial Version 5)”*.

## Discussion

The aim of this study was to analyze gender differences in clinical outcomes and psychological status of patients with 2019-nCoV infection. The results demonstrate that the smoking rates of male patients was significantly higher than female patients, which is consistent with smoking demographics in China. Smoking is considered a critical factor in the progression of 2019-nCoV infection (8). In addition, the participants' laboratory results showed that the rate of increased C-reactive protein levels was significantly higher in male patients than in female patients. C-reactive protein is an important indicator of inflammation and is currently believed to be a crucial factor in the prognosis of 2019-nCoV infection (8). As pneumonia progresses, C-reactive protein levels are relatively higher in the most severe cases (9, 10). However, no statistically significant differences were discovered in white blood cell counts, lymphocyte counts, and procalcitonin levels between genders in the present study. Based on the above findings, it was indicated that smoking might be the cause of the higher rate of dry cough among male patients in this study and related to the progression to critical illness of more male cases than female cases. As such, smoking cessation is an effective measure in the treatment of 2019-nCoV infection.

The most salient finding in this study is that anxiety and depression were more prevalent in the female cohort compared to the male cohort, while the overall clinical classification and MuLBSTA score of male patients was higher than female patients. When faced with a sudden public health emergency, people are generally prone to develop psychological issues, such as tension, anxiety, panic, and pessimism, among others. Research on the SARS pandemic found that, while it was initially ignored, people's emotional responses to the event included fear, annoyance, complaints, and anxiety; these responses progressively developed into depression, loneliness, helplessness, hopelessness, and sadness (11, 12). Anxiety and depression may decline over time; however, some of the symptoms may persist throughout the disease process, affecting the effectiveness of treatment and follow-up recovery (13). Results of an analysis of gender differences in the mental health of patients during the SARS pandemic show that the severity of psychological problems among female patients was significantly higher than in male patients (14). Moreover, female gender was identified as the most potent indicator of post-traumatic stress symptoms after the 2019-nCoV outbreak (15). The results of this study are consistent with previous studies, which show that females have experienced greater psychological problems than males during the 2019-nCoV pandemic. Thus, greater attention should be paid to the mental health of the female patients during the pandemic.

Previous studies have shown conflicting results regarding gender differences in the severity of 2019-nCoV infection. A recent meta-analysis proposes that male gender may be a predictor of more severe 2019-nCoV infection but not of mortality (16). An analysis of 78 patients with 2019-nCoV infection in Anhui Province found that male patients accounted for more severe and critical cases compared to female patients (9), which may be closely associated with the higher rate of smoking among Chinese men. Additionally, the MuLBSTA score has been reported to be a strong predictor of the risk of death in patients with viral pneumonia (7). Some studies have revealed that male 2019-nCoV infection cases, especially among elderly patients with underlying health problems, have a higher mortality rate compared with female cases (17). In the present study, male patients had higher MuLBSTA scores compared with female patients, and all three deaths that occurred during the study were male. These findings confirm the suggestion that male gender plays a critical role in the severity and mortality of 2019-nCoV infection.

It was not unexpected to find that the mental health status of the study patients was not parallel to the severity of their infection and or risk of mortality, since the mental health of the less severe patients is usually ignored by front-line clinical staff. Therefore, aside from the treatment of pneumonia, more psychological interventions should be provided for female patients, and smoking cessation interventions and advanced therapeutic treatments should be provided to male patients in clinical care.

There are not without some limitations in this study. First, the data was taken from the inpatients of 2019-nCoV infection in a single hospital, and 2019-nCoV infection presenting with mild symptoms was not included as this population was either quarantine at home or in the mobile field hospitals, therefore some finding might not by fully representative the whole cohort of 2019-nCoV infection. Secondly, due to the termination of the researchers' support work in Wuhan, some few patients were not fully tracked till the end, so the disease outcomes of these patients were not completely accurate. In a follow-up study, the research team plans to collect more data from more hospitals, and extend the investigation content. Multivariate logistic regression analysis in the single gender group will also be applied to provide a stronger evidence base for the prevention and control of 2019-nCoV infection.

## Conclusion

Results of this study indicate the need for the implementation of different interventions and nursing measures based on gender in the treatment of 2019-nCoV infection in hospitals. Generally, female patients experienced more severe psychological issues due to higher levels of anxiety and stress; thus more attention might be paid to the psychological counseling and care of these female patients. Comparatively, the psychological status of male patients appeared to be less intense, but their general condition was more severe, particularly in elderly patients with a history of chronic disease and smoking. This population is, therefore, the focus of clinical care and is likely to require increased monitoring and respiratory support. Male patients should be encouraged to quit smoking as soon as possible in order to reduce the risk of severe pneumonia during the 2019-nCoV infection.

## Recommendation

1. This study sheds light on the gender differences in psychological status and reveled that mental health level was not parallel to the severity degree of disease.
2. The study discovered that female experienced sever psychological issues than male, thus more attention should be paid to the psychological counseling and care of these female during the 2019-nCoV infection.
3. The study revealed that male patients had a higher level of mortality rate and disease severity degree than those of female patients, particularly in the elderly patients with a history of chronic disease and smoking.

## Data Availability Statement

The original contributions presented in the study are included in the article/supplementary material, further inquiries can be directed to the corresponding authors.

## Ethics Statement

The studies involving human participants were reviewed and approved by the study was conducted in accordance with the Declaration of Helsinki and approved by the Ethics Committee of Xiangya Hospital of Central South University (Approval No. 202003049). The patients/participants provided their written informed consent to participate in this study.

## Author Contributions

HH, FZ, XP, GD, XC, and LP contributed in conception, study design, coordination of data collection, and acquisition in data. HH, FZ, XP, and GD were responsible for interpretation of data, drafting, writing, and finishing the manuscript. All authors contributed to the article and approved the submitted version.

## Conflict of Interest

The authors declare that the research was conducted in the absence of any commercial or financial relationships that could be construed as a potential conflict of interest.
